# Can human schistosomiasis mansoni control be sustained in high-risk transmission foci in Egypt?

**DOI:** 10.1186/s13071-015-0983-2

**Published:** 2015-07-16

**Authors:** Hala Elmorshedy, Robert Bergquist, Nadia Emam Abou El-Ela, Safaa Mohamed Eassa, Elham Elsayed Elsakka, Rashida Barakat

**Affiliations:** Department of Tropical Health, High Institute of Public Health, Alexandria University, Alexandria, Egypt; Ingerod, Brastad, Sweden & University of Basel, Basel, Switzerland; Department of Pediatric, Faculty of Medicine, Alexandria University, 165 Alhorreya Avenue, Alexandria, Egypt

**Keywords:** *S mansoni*, Control, Praziquantel, Nile Delta, Egypt

## Abstract

**Background:**

Control of human schistosomiasis remains a longstanding issue on the agenda of the Egyptian Ministry of Health and Population (MOHP). Substantial impact on morbidity and prevalence of *S. mansoni* was widely reported after the National Schistosomiasis Control Program (NSCP) extended selective treatment with praziquantel (PZQ) to the Nile Delta in 1992 and upgrading this approach to mass drug administration (MDA) in 1997. Disease elimination, however, eludes NSCP as the micro-level includes many high-risk foci that sustain transmission, which has not been subjected to investigation.

**Methods:**

The study included five high-risk Nile Delta villages situated in the Kafr El-Sheikh Governorate. The total sample size amounted to 2382 individuals of both sexes and all ages. Diagnosis was based on four Kato-Katz slides from two consecutive stool samples. Data were investigated using SPSS, comparing proportions with the Chi square test and means with the Student *t* test, while strength of the associations were subjected to Odds Ratio (OR) analysis.

**Results:**

The overall prevalence of schistosomiasis in the study area was found to be 29 %, while the mean geometric mean egg count (GMEC) was low (66.78 ± 4.4) indicating low intensity of infection. The mean village prevalence rates ranged from 16.5 % to 49.5 % and the GMECs from 35.2 to 86.2 eggs per gram (EPG) of stool. The difference of prevalence between villages was statistically significant at P < 0.05, and the prevalence was significantly higher among males than among females, P < 0.05, OR =1.4 and 95 % CI (1.16-1.60). Infection peaked in the next youngest age group (5- ≤ 10 years of age) at an average prevalence of 50.8 % with the GMEC reaching 209 EPG of stool in the village with the highest prevalence. The average prevalence and GMEC among children <5 years were 20.6 % and 92.7 EPG, respectively.

**Conclusion:**

Transmission of *S mansoni* in high-risk areas in the Nile Delta remains uninterrupted calling for improved, more comprehensive control strategies. Further investigations are needed to find out whether these results are due to inefficacy of PZQ, surviving immature worms or drug resistance.

## Background

The signing on 31 January 2012 of the London Declaration by international politicians, heads of pharmaceutical businesses and health-related, non-governmental organizations (NGOs) represents a breakthrough in the fight against the world’s neglected tropical diseases (NTDs) [1]. This commitment, based on a plan presented by the World Health Organization, offers hope that as many as 10 diseases, among them schistosomiasis, can be controlled or eliminated as early as by the end of the current decade [2].

Schistosomiasis is caused by a long-standing infection with trematode worms of the genus *Schistosoma*. Humans get infected during water contact through the release of the infectious schistosome cercarial stage from the parasite’s intermediate snail host. The cerariae grow into adult worms, which excrete a large number of eggs that will eventually infect the snail when hatched in water after being delivered there by faeces (*Schistosoma mansoni* alternatively *S. japonicum*) or urine *S. haematobium* (additional species exist but are uncommon). Transmission is sustained by lack of clean water and sanitation, which explains why this is a disease of poverty [3]. The sequelae of immune-mediated responses to schistosome eggs stranded in the human host varies according to schistosome species. Untreated, the disease eventually produces fibrosis and obstructive manifestations due to damage of the liver (in *S. mansoni* infections) or the bladder (in *S. haematobium* infections) [4].

Human schistosomiasis is endemic at variable rates in 77 countries in tropical and subtropical regions of the world, where the most recent estimates suggest that more than 250 million people are infected resulting in a global burden of 3.3 million disability-adjusted life years (DALYs) [5, 6]. Various drugs and other measures have been used to control schistosomiasis. However, shortly after its discovery in the1970s [7], and follow-up clinical trials [8], praziquantel (PZQ) replaced all other anti-schistosomal drugs and is now the corner stone of schistosomiasis control programmes the world over. In the late 1990s, treatment strategies shifted from selective treatment to mass drug administration (MDA) that means treatment of all individuals living in endemic areas regardless of infection status [9].

Approximately, 12.7 million infected individuals are clustered in the Middle East and North Africa (MENA) region and Egypt’s share of the burden is about 7.2 million [10]. Both *S. mansoni* and *S. haematobium* species are represented with the former species currently increasing in importance and now dominating in the Nile Delta [11]. *S. mansoni* existed only sparsely along the Nile previously and did not become common there until the 20th century, a change ascribed to the ability of the *S. mansoni* snail host *Biomphalaria* to survive in the less oxygenated water resulting from intensified irrigation practices [12, 13] followed by the Aswan High Dam construction [14]. The replacement of *S. haematobium* with *S. mansoni* in the Nile Delta was almost complete already 15 years ago [15], and it is today unusual to find cases of urinary schistosomiasis in this part of Egypt. Several studies have documented this transformation and the appearance of new foci favoring transmission of *S. mansoni* [16–19].

Regular control of schistosomiasis has been on the agenda of the Egyptian Ministry of Health and Population (MOHP) since 1922. Activities were strongly upgraded in 1976 through the organization of the National Schistosomiasis Control Project (NSCP), which started control measures in the most endemic areas in Upper and Middle Egypt. In 1992, activities were extended to all endemic areas including the Nile Delta and in 1997, following WHO’s recommendation, MDA was instituted throughout the country [20]. The impact was rapid and substantial with *S. haematobium* prevalence in Middle and Upper Egypt falling from an average 29.3 % in 1977 to less than 3 % by the end of the 1990s [20, 21] followed by an even lower rate for *S. mansoni* (1.5 % average prevalence) in the Nile Delta by 2006 [22]. However, the long-term results were not as positive everywhere. Baseline data collected for a study before the first NSCP intervention in 1992 showed an average prevalence of 39.3 % of schistosomiasis mansoni with an intensity of infection as measured by the geometric mean egg count (GMEC) of 72.9 eggs per gram (EPG) in Kafr El-Sheikh, a governorate in the northern part of the Nile Delta [19, 23]. In 1993, after a period of active case finding and selective chemotherapy of infected individuals for two successive years, a significant drop in prevalence and intensity of infection based on single stool examinations using the Kato-Katz method was initially achieved, both in high- and low-risk areas [23, 24]. However, in spite of the ongoing control activities this trend was not sustained. Although prevalence rates and GMECs remained below the baseline data, an upward trend was observed in high-prevalence villages in 1997 [24, 25].

In view of more than 20 years of continuous treatment with PZQ in the Nile Delta with elimination of morbidity but stalling prevalence reduction, it was felt necessary to revisit the situation and investigate the recent outcome of long-term impact of MDA in a sub-regional, endemic high-risk area.

## Methods

As per the Egyptian MOHP control strategy of human schistosomiasis, and in accordance with the WHO’s recommendations [20], PZQ MDA was instituted in 1997. The rational was to scale up the application of MDA aiming to control transmission rather than morbidity. PZQ MDA is recommended if the prevalence of schistosomiasis exceeds 3 % compared to 20 % according to the WHO recommendations [11, 20–22]. Prevalence data are based on annual screening of school children carried out by the local Rural Health Units’ teams and further confirmed by examining a random sample of all ages carried out by independent sentinel mobile teams assigned by the regional health districts. Accordingly, PZQ MDA was offered to all individuals 6 years and above. In 2011, all inhabitants of the villages included in our study received PZQ in a single oral dose of 40 mg/kg couple of months before the inception of the study. Before 2011, review of the official reports of endemic disease departments of health directories both in the Metobas District and in the Kafr El-Sheikh Governorate by the authors revealed that these villages were subjected to PZQ MDA annually since 2007.

### Study site and population

**Fig. 1:**
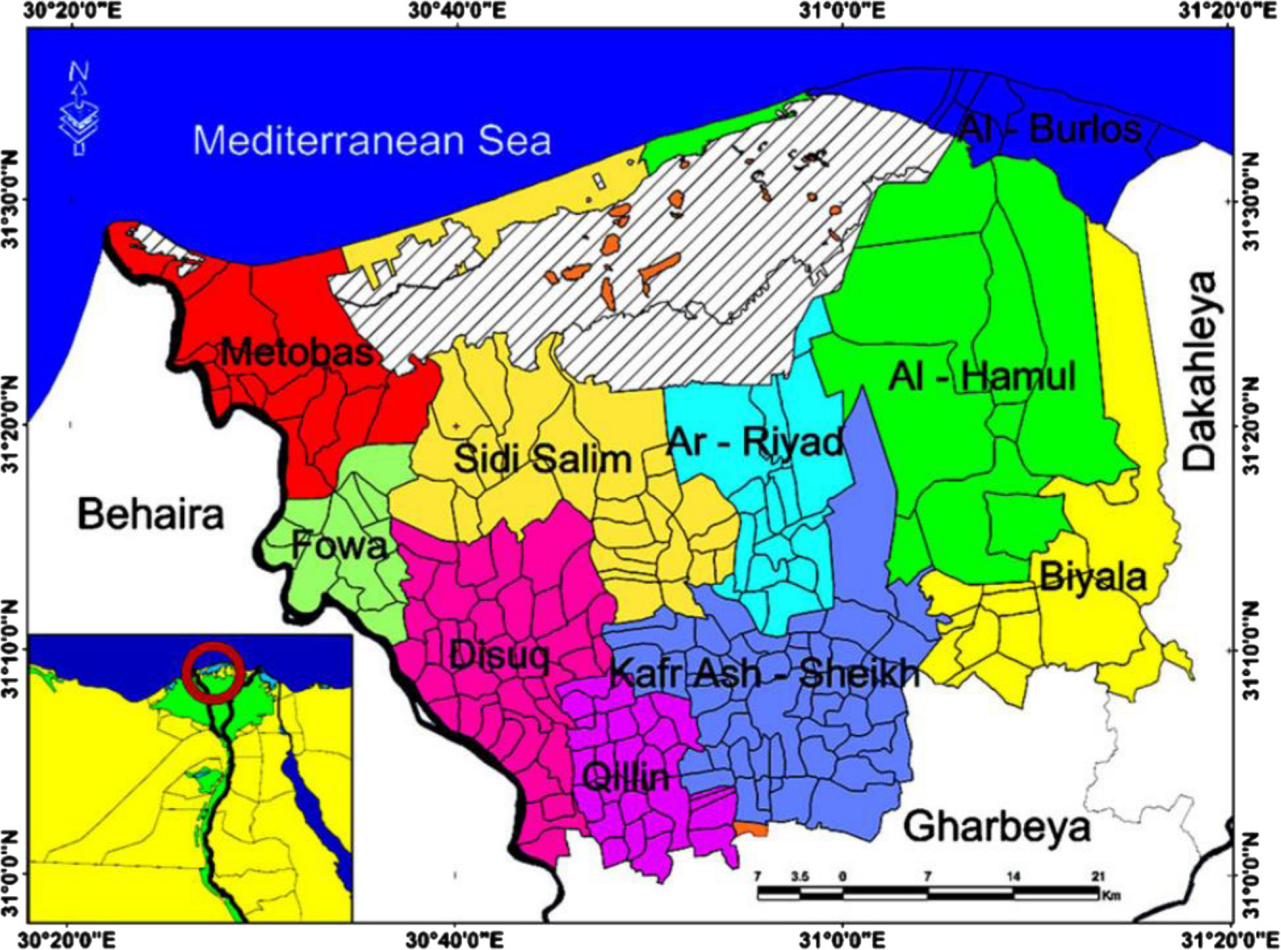
The districts of Kafr El-Sheik Governorate in the Northern Nile Delta

In 2011, after a period of large-scale PZQ treatment carried out by the MOHP in the Nile Delta, a cross-sectional study on schistosomiasis prevalence was undertaken in the Al-Gezeera Al khadra and Arab Al Mahdar villages, including the three small villages/ezbas of Al Ouidat, Abou Zeid, and Abou Farag, Metobas District of the Kafr El-Sheikh Governorate (Fig. 1). These villages were selected for the study because of their continued high prevalence of schistosomiasis mansoni. All the villages are situated in the northern part of the Nile Delta bounded by Rosetta branch of the Nile River in the West and the Mediterranean Sea in the North. The location of these villages at the far north part of the Rosetta branch (i.e., end of the stream) provides flourishing breeding habitat of the snail intermediate host of *S. mansoni* because of the slow water currents and the abundant vegetation in canals and drains. Of note, human water contact is intense in these villages since fishing and rice cultivation are the occupations of the majority of people. Furthermore, the inhabitants of these villages depend partly on public tap water, whereas they still use canal water intensively for domestic activities. Sanitary sewage disposal is also lacking.

### Approach

Schsistosomiasis is a seasonal disease, where most infections are acquired during the hot months of summer. The field work of this study was conducted in 2011 during the period from April through June where disease transmission occurs at an appreciable level. We first conducted a house-to-house census survey inviting all age groups to participate in the study. The number of houses included in the study varied: 37 in Abou Farag, 38 in Al Ouidat, 56 in Abou Zeid, 66 in Arab Al Mahdarand 74 in Al Gezeera Al khadra. The total number of participants in the study amounted to 2382 individuals. The distribution of study subjects per village was proportional to the number of houses. All the households provided demographic information, such as age and sex, that was collected and organized by trained data collectors.

### Diagnosis

All participants and guardians of young children were requested to provide two stool samples in two consecutive days, collected at the same time each day. These samples were processed in the field using the Kato-Katz technique [26]. Briefly, two slides each of 41.7 mg of stool were prepared from each sample and day. The egg counts of the four slides were averaged and the outcome used for the analysis. To insure validity and reliability of the parasitological data, 10 % of randomly selected slides were re-examined and the EPG recounted. In addition, any slide with only one egg was also rechecked by an experienced parasitologist.

### Statistical analysis

Data were analyzed using SPSS, version 20.0 (IBM, New York, NY, USA). All EPG values were transformed into log 10 of the EPG +1 to allow for the zero counts and the GMEC was computed as the anti-log 10 of the mean of the log10 egg count. This was used because in areas where repeated therapy interventions have taken place, the distribution of the intensity of infection measured by EPG has the tendency to become right-skewed, i.e., in the majority of cases, there are only a few or no parasites left after treatment, while a few individuals showed relatively large parasite burdens, a tendency best described by considering their GMEC. We used odds ratio (OR) to express the strength of association between dichotomous variables and the Pearson’s Chi square test to compare between proportions. The independent sample *t*-test was used to compare the intensity of infection as measured by GMEC with regard to villages and age categories. All tests were two-sided with the *p* = 0.05 level for statistical significance accepted as the cut-off value.

### Ethical approval

The study was reviewed and approved by the Ethical review Board, High Institute of Public Health, Alexandria University, Egypt.

## Results

Out of the 2382 individuals interviewed, 1493 (including both sexes and all ages) provided stool samples. Table 1 demonstrates the prevalence of *S. mansoni* infection according to village and gender. The overall prevalence was 29 % with the highest value in Arab Al Mahdar (45.9 %). With the prevalence rates for the other villages ranging from 16.5 % to 32.3 %, it is evident that all the study villages must be considered as having moderate schistosomiasis risk. The difference in prevalence between villages was statistically significant at the p < 0.05 level. With respect to gender, the overall prevalence of infection was significantly higher among males than among females: P < 0.05, OR =1.4 and 95 % CI (1.164-1.6). Although the prevalence of infection was higher among males than among females in all villages, the difference was only statically significant in Arab Al Mahdar, Al Ouidat and Abou Zeid, the three villages with the highest prevalence values.

**Table 1:** Prevalence of *Schistosomamansoni* infection with respect to village and gender

Area (Ezba)	Total population			Males		Females	
	Number examined	Number positive	% positive	Number positive	% positive	Number positive	% positive
Arab Al Mahdar	377	173	45.9	93	51.4	80	40.8^a^
Al Ouidat	O260	84	32.3	47	38.5	37	26.8^a^
Abou Zeid	204	49	24	32	29.9	17	17.5^a^
Al Gezeera Al Khadra	415	88	21.2	42	23.1	46	19.7
Abou Farag	237	39	16.5	20	20.2	19	13.8
Total	1493	433	29^b^	234	33.9	199	24.8

^a^Statistically significant at p <0.05

^b^Differences between the village prevalence rates were statistically significant (*X* ^2^ = 86.42, *p* = 0.00)

**Fig. 2:**
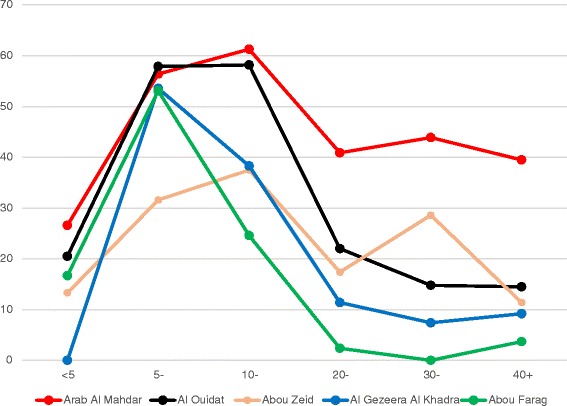
*S. mansoni* prevalence with regard to age and village

When people are divided into 10-year ranges according to age, it can be seen that the cases in all villages were clustered in the range between 5 and 20 years of age, followed by a considerable reduction of cases among older people (Fig. 2). However, an increased prevalence for 30-40 and 40-50 year olds were also found in Abou Zeid and Arab Al Mahdar, respectively.

In Table 2, the infection pattern was analyzed from the viewpoint of three age categories: <5, 5 - ≤10 and >10. It is again obvious that the burden of infection is the highest among young people, in particular in the group aged 5 - ≤10, where there was about a two-fold increase in prevalence as compared to those in the >10 age group (OR = 1.9, 95 % CI(1.61-2.62)) and 2.5-fold compared to children below 5 (OR = 2.5, 95 % CI (1.79 – 3.47)). It is notable that across the villages, the difference in prevalence was not statistically significant P > 0.05 for the first two age categories (those below10 years of age).

**Table 2:** Prevalence of *Schistosomamansoni* infection according to age group

Age	<5		5 - ≤10		>10	
Village	Number	% positive	Number	% positive	Number	% positive
Arab Al Mahdar	64	26.6	55	56.4	256	48.3
Al Ouidat	44	20.5	38	57.9	178	29.9
Abou Zeid	30	13.3	38	31.6	136	24.3
Al Gezeera Al Khadra	5	0.0	28	53.6	381	19.2
Abou Farag	24	16.7	32	53.1	181	9.9
Total	167	20.4	191	50.8	1135	26.6
*X* ^2^	---	3.912	----	7.219	--	100.048
P		0.074		0.272		0.00

**Fig. 3:**
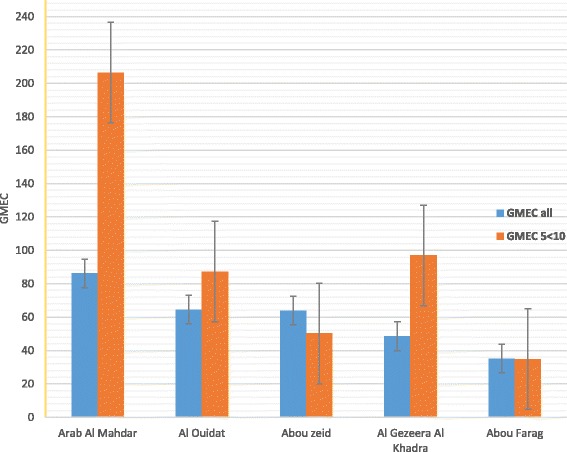
Geometric means of egg counts among children 5- < 10 years versus total population with regard to village

Table 3 and Fig. 3 demonstrate that the intensity of infection (GMCE) increased as prevalence increased. The overall GMEC was 66.7 ± 4.11, while it was 92.7 and 95.6 for children <5 and 5 - ≤10, respectively. In comparison, GMEC was only 57.3 in the group >10 years of age. Intensity of infection was significantly higher among children ≤ 10 years of age,

**Table 3:** Intensity of *Schistosomamansoni* infection according to age group

Age group Village						
	<5		5 - ≤ 10		>10	
	Positive	GMEC^a^	Positive	GMEC^a^	Positive	GMEC^a^
Arab Al Mahdar	17	128.9 ± 4.9	31	209.7 ± 5.1	125	63.1 ± 3.4
Al Ouidat	9	42.5 ± 3.2	22	90.01 ± 5.2	53	63.7 ± 4.3
Abou Zeid	4	109.4 ± 8.2	12	52 ± 3.5	33	67.2 ± 3.0
Al Gezeera Al Khadra	0	--	15	99.80 ± 6.2	73	44.0 ± 3.5
Abou Farag	4	111.6 ± 3.9	17	36.60 ± 3.4	18	29.4 ± 3.6
Total	34	92.7 ± 4.7	97	95.6 ± 5.26	302	57.3 ± 3.62

^a^Mean geometric mean egg count ± SD

GMEC was significantly higher among children ≤ 10 years,*P* = <0.05

*P* = <0.05. This relation was more prominent in the village with the highest prevalence, yet it was maintained in nearly all the communities.

## Discussion

Schistosomiasis control based on MDA using PZQ in Egypt has been exceptionally successful, both with regard to morbidity and to prevalence [20, 22]. However, these reports are country- or region-wide, thus neglecting the potential impact due to focal high-risk areas, which become masked by results from larger areas in other parts of the country with currently negligible prevalence rates. The presence of local situations, such as Kafr El-Sheikh, where the probability of immature infection is high due to numerous patches with increased levels of transmission, calls for modification of local control strategies during the implementation phase of chemotherapy taking into account the fact that PZQ is ineffective against the schistosomula and immature worms [27]. Possible modifications include temporally closer PZQ rounds or use of drug combinations including a complementary artemether derivative [28].

Comparison of the results of the present study with those from the time just before the initial active intervention (the baseline data) [29], clearly demonstrates that the ongoing chemotherapy programme remains effective with respect to morbidity. Prevalence previously peaked in adolescents (16-20 years of age), while the GMEC was lower, probably thanks to long-term availability of PZQ [29]. The low prevalence and low intensity of infection among older age groups versus an almost 2.5-fold increase in prevalence and a fairly high GMEC in children below 10 seen in the present study can be attributed to partial immunity [30]. A decrease in prevalence and intensity of infection among older individuals would then be expected, yet this decrease may not reflect an actual decrease in the prevailing strength of transmission. In reality, it is possible that local transmission could actually be on the increase since prevalence and GMEC in children were higher in our study than reported previously [31]. However, although the moderate GMEC rates seen here (Table 3) indicate that ongoing interventions are successful with regard to morbidity, Table 2 and Fig. 2 reveal that prevalence rates among children are unacceptably high reflecting continued high levels of transmission. Thus, the main reason for the failure of elimination of schistosomiasis locally seems to be the inability of current control activities (MDA with PZQ) to stop transmission, even when regularly repeated.

Following two rounds of PZQ selective treatment in Kafr El-Sheikh, the prevalence rates of the high-risk villages approached that of the low-risk ones: 20.5 % and 19.5 %, respectively, as compared to 44.9 % and 33.5 %, reported for them at the baseline [31]. In addition, two previous studies in this governorate reported lower prevalence rates (29 % and 45 %, respectively) among primary school children than our present study. In both these studies, the GMEC values were also low (62 and 58, respectively) [28, 32]. Despite the fact that both these studies report good effect on morbidity with prevalence data at a lower boundary than our study, there was no influence on transmission.

The results from a majority of the study villages corroborate the well-known picture of peak prevalence and intensity of infection among 5-10 years old young children (Figs 2 and 3, Tables 2 and 3) followed by significantly lower values in adolescence and old age. In Arab Al Mahdar and Abou Zeid, however, there was no such marked reduction of prevalence after the initial peak. Although both these villages showed a similar development over the age groups, the former showed considerably higher prevalence rates for all age groups compared to the latter. On the other hand, the pattern of schistosomiasis prevalence among children less than 10 years old (the two youngest groups tested) was similar in all the villages studied with no statistically significant inter-village difference (Tables 1 and 2). Observed variations in the overall prevalence between the villages are therefore exclusively contributed by older individuals, particularly in Arab Al Mahdar and Abou Zeid, which emphasizes failure of instituted control measures. However, this should be seen against the background of the overall high prevalence that would have been lower, had transmission been less pronounced.

The potential underlying factors for the observed rebound in infections in spite of repeated PZQ treatment schedules are several. Concerns regarding the emergence of schistosome strains with reduced susceptibility or tolerance to PZQ has been expressed [33]. The argument, whether this represents a selection of resistant worm populations under drug pressure or incomplete cure [34] is still an open question. However, extensive application of PZQ for almost 40 years in Upper and Middle Egypt and for more than 20 years in the Nile Delta should have imposed strong selection of the parasite populations in all of Egypt. There are reasons to believe that continuous drug pressure for this long should have had an effect by now and there is evidence pointing in this direction, e.g., microsatellite analysis has shown presence of a single *S. mansoni* genotype in naturally infected intermediate host *Biomphalaria alexandrina* snails collected from water channels in the Nile Delta [35]. Moreover, reduction in genetic diversity of a Tanzanian schistosome population was reported after only one round of treatment [36]. Naturally selected drug resistance has also been reported for oxamniquine in Brazil [37].

Apart from the role of stage-selective drug action, as that exerted by PZQ, cure rates do not reach 100 % but rather range from 80 % to 90 % as noted in Kafr El-Sheik [32]. There are also other factors having to do with socio-cultural and environmental issues as well as the quality of health service operations at the peripheral level. Potential complacency with regard to repeated chemotherapy and follow-up testing is yet another problem that needs to be investigated. The combined effect of all these factors is difficult to estimate, but there will be no let-up in transmission as long as parasite eggs continue to be excreted directly into the field.

Whether the observed high prevalence rates among children in our study is due to strong transmission resulting in reinfection, development of surviving schistosomula into adult worms or reduced susceptibility of adult worms to PZQ should be considered in addition to the ongoing regime, especially for the high-risk areas. Combining chemotherapy of PZQ with, for example, artemether that exclusively affects the immature worm should contribute to a better cure rate by covering the full spectrum of the parasite development in the definitive host. Such combination therapies have been used in Kafr El-Sheikh with impressive results [28]. Following one round of combined treatment, prevalence was reduced from 30 % to 6.8 % and incidence of new infections fell to 2.7 %. Still, elimination based on chemotherapy alone, given the presence of remote high-risk areas, will be difficult to achieve, so other measures as socio-cultural development, environmental sanitation and functioning public health services are necessary adjuncts.

## Conclusion

- PZQ is the solution to morbidity in endemic areas but only if regularly repeated. Continued transmission after PZQ treatment is well-known, but the long-term consequences of this fact have not been given appropriate attention.
- Focal transmission of *S mansoni* in the Nile Delta remains uninterrupted calling for improved, more comprehensive control strategies. PZQ treatment should remain the cornerstone of control, but elimination strategies need additional input, e.g., snail control,complimentary drugs, upgraded sanitation and/or improved public health service capable to deal with potential complacency with regard to repeated chemotherapy rounds.
- As long as some patients remain infected, eggs will continue to be excreted into the field assuring continued transmission. The observed rebound of schistosomiasis in spite of long-term, repeated PZQ treatment in the Nile Delta is therefore cause of grave concern. Further investigations are needed to find out the reason(s) for the current failure of up-to-date control strategies in the Nile Delta

## Abbreviations

NGO: Non-governmental organizations
NTDs: Neglected tropical diseases
DALYs: Disability-adjusted life years
PZQ: Praziquantel
MDA: Mass drug administration
MENA: Middle East and North Africa
MOHP: Ministry of Health and Population
NSCP: National Schistosomiasis Control Project
GMEC: Geometric mean egg count
EPG: Egg per gram of stool
